# Improving Diffusion in Collagen Hydrogels for 3D Culture of Rat Cardiac or Dermal Fibroblasts via Magnetically Actuated Vibrating Microparts

**DOI:** 10.3390/gels12030225

**Published:** 2026-03-10

**Authors:** Kenji Inoue, Zhonggang Feng, Yuta Higashiyama, Toshifumi Kawaguchi, Takehiro Matsuura, Masaharu Abe

**Affiliations:** Graduate School of Science and Engineering, Yamagata University, Yonezawa 992-8510, Japan; yutahigashiyama3@gmail.com (Y.H.); kawa@yz.yamagata-u.ac.jp (T.K.); takehiro.1027@icloud.com (T.M.); t243276m@st.yamagata-u.ac.jp (M.A.)

**Keywords:** iron particles, magnetic activation, microtubes, cell culture, medium diffusion, internally distributed driving source

## Abstract

Ensuring efficient nutrient delivery and waste removal within the interior of three-dimensional (3D) cultures remains a major challenge in tissue engineering. Here, we demonstrate a proof-of-concept methodology that creates internally distributed driving sources to enhance diffusion and perfusion within 3D constructs. Iron microparticles or iron-containing microtubes were incorporated into collagen gels used for the 3D culture of dermal or cardiac fibroblasts, and cyclic dynamic magnetic fields were applied to the constructs. Oscillatory motion of the iron particles enhanced diffusion within the gels, as evidenced by increases in the fast diffusion coefficient of more than threefold and the slow diffusion coefficient of more than tenfold under conditions suitable for cell culture. In cardiac fibroblast cultures, this enhancement significantly increased proliferation by approximately twofold and reduced cytotoxicity by half compared with controls. In contrast, no significant effects were observed in dermal fibroblast cultures. Cyclic compression of microtubes within the collagen gels induced by dynamic magnetic fields primarily resulted in cellular morphological changes, including a reduction in cell area to approximately 0.8-fold of the control values, increased cell polarization with the cellular aspect ratio rising from 1.4 to 1.9, and preferred cell orientations either parallel or perpendicular to the microtube axis. Together, these results suggest that this methodology has the potential to be developed as an effective strategy for improving diffusivity in 3D metabolic environments and for promoting angiogenesis in hydrogel-based cultures.

## 1. Introduction

Three-dimensional (3D) culture systems are fundamental and often indispensable for generating physiologically relevant tissue equivalents in tissue engineering and regenerative medicine. These models are critical for replicating the complex cell–cell and cell–matrix interactions that govern tissue development, function, and disease progression in vivo, which cannot be adequately captured in conventional two-dimensional monolayer cultures. Hydrogels have emerged as a dominant class of scaffolds for 3D tissue construction owing to their biomimetic, water-swollen polymer networks that closely resemble native extracellular matrices. Their relatively high diffusivity eases nutrient delivery and waste removal, while their excellent biocompatibility and tunable mechanical properties support cell viability and phenotype. Moreover, the spatiotemporally controllable gelation of many hydrogels—often triggered by physical or chemical cues—allows precise fabrication of 3D constructs. Collectively, these attributes make hydrogels uniquely advantageous for implementing a wide range of biofabrication strategies, from top-down molding of bulk constructs to bottom-up assembly of modular tissue units, thereby driving innovation in the creation of complex, functional tissues [1,2,3,4,5].

Despite these advantages, a major challenge remains: ensuring efficient nutrient delivery and waste removal within the interior regions of increasingly large 3D hydrogel constructs, which is critical for maintaining long-term cell viability and function [6,7,8]. The ultimate biomimetic solution to this mass transport limitation is the development of perfusable, capillary-like vascular networks within engineered tissues. To this end, a variety of microvascularization strategies have been explored, including biological approaches that exploit the intrinsic ability of endothelial cells to form vascular structures through sprouting and tubulogenesis. In parallel, numerous engineering-based strategies have been developed, encompassing templating, modular assembly, microfabrication, three-dimensional bioprinting, rapid prototyping, and hybrid techniques that integrate multiple fabrication modalities [7,8,9]. Nevertheless, the fabrication of tissue-scale, stable, and functionally perfusable microvascular networks within 3D hydrogels remains an unresolved engineering challenge. This limitation continues to hinder the translation from small, laboratory-scale constructs to large, implantable, and self-sustaining tissue equivalents, which remains one of the foremost goals in the field [6,8,10,11,12].

In light of these challenges, we propose a complementary strategy that enhances hydrogel diffusivity by embedding oscillating microparts powered by externally applied, non-contact, reciprocating fields, such as magnetic or electrical fields. This concept builds on the observation that, even in the absence of microvascular structures, 3D hydrogel constructs still perform as effective tissue models [1,2,13,14] and have demonstrated substantial utility in clinical and drug-screening applications [15,16,17,18]. Thus, while the incorporation of microvasculature is highly desirable, it is not an absolute requirement for all 3D hydrogel culture contexts.

Enhancing diffusivity within hydrogels offers three major benefits for 3D cell culture. First, increased diffusivity improves the metabolic microenvironment experienced by encapsulated cells. Second, the motion of embedded microparts can introduce localized mechanical stimuli—such as microstrain or stress—that may elicit cellular mechanotransductive responses. Third, specific types of activatable microparts, including the microtubes developed in this study, may promote endothelial cell sprouting and angiogenesis, thereby offering a potential pathway toward the formation of capillary-like networks within hydrogels.

Based on these considerations, we embedded collagen hydrogels with two types of iron-based microparts: simple iron microparticles and iron-containing microtubes. Both micropart types were activated using an externally applied, reciprocating magnetic field. In this study, we evaluate the fundamental physical properties of these micropart-embedded 3D collagen gels, as well as preliminary outcomes of their application in 3D cell culture.

## 2. Results and Discussion

### 2.1. Enhanced Diffusion by Iron Particle Oscillation in Collagen Gels

**Figure 1:**
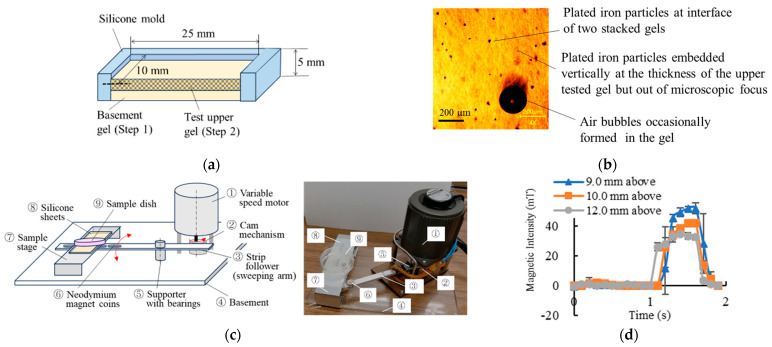
(**a**) Schematic illustration of the fabrication of a two-layer 3D culture test gel using a silicone mold via a two-step process. (**b**) Representative photograph of plated iron particles embedded in the test gel. Iron particles are distributed within the upper gel layer, while most precipitate at the interface between the two gel layers. (**c**) Schematic illustration and photograph of the sweeping device used to apply a cyclic dynamic magnetic field to culture gels placed on the stage. Red arrows indicate the direction of movement. (**d**) Magnetic field intensity profiles (n = 5 cycles) within the culture dish measured at three different distances above the coin magnets mounted on the sweeping arm.

Collagen gels were employed as engineered tissue models to evaluate diffusion enhancement induced by embedded oscillatory microparts. Figure 1a illustrates the structure of the experimental platform, consisting of a two-layer collagen gel with gold-plated iron microparticles embedded within the upper layer. The average particle diameter was 50.0 ± 7.0 μm. After formation of the upper test gel layer (Figure 1b), the particles were distributed throughout the gel thickness, although the majority sedimented near the interface between the upper and lower layers. To induce oscillatory motion of the embedded iron microparticles, we developed a dynamic magnetic actuation system (Figure 1c; Supplementary Video SI-v1), hereafter referred to as the “sweeping device.” The magnetic field intensity at the sample stage was characterized at three distances—9.0, 10.0, and 12.0 mm above the coin magnets mounted on the sweeping arm—as shown in Figure 1d. Oscillatory particle motion within the collagen gel (Supplementary Video SI-v2) was directly observed using an inverted microscope (IX71, Olympus, Tokyo, Japan). At a magnet–sample distance of 10.0 mm, the particle oscillation amplitude was measured to be 7.0 ± 5.0 μm (n = 10).

The spatial scale of the hydrodynamic perturbation induced by particle oscillation can be estimated by the Stokes boundary layer thickness, δ ≈ 370 μm at an oscillation frequency of 1.0 Hz. We found that the average interparticle spacing was approximately 200 μm when 2.0 mg of iron microparticles was embedded in the upper gel layer. Considering the diffusion barrier imposed by the collagen network, this spacing was deemed appropriate for effective hydrodynamic interaction between neighboring particles. Accordingly, a loading of 2.0 mg iron microparticles was selected for subsequent cell culture experiments.

**Figure 2:**
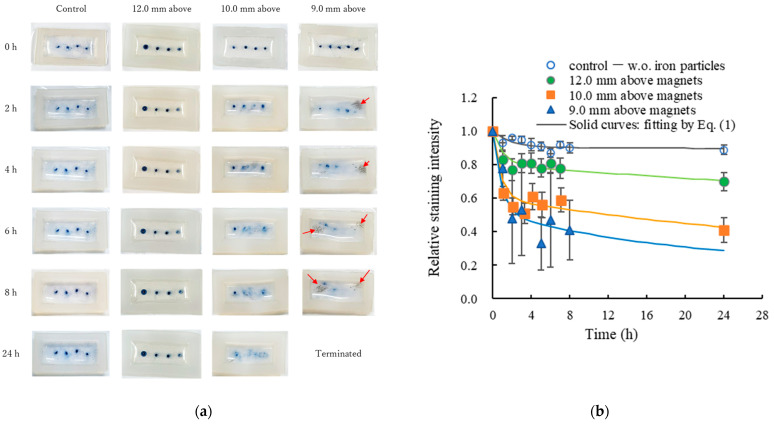
(**a**) Time-lapse images showing ink diffusion in test gels without magnetic actuation (control) and with magnetic actuation as gels positioned at three different distances above the magnetic sweeping arm. Red arrows indicate where particle aggregation and migration occurred. (**b**) Results of ink diffusion (n = 4) under different magnetic field intensities corresponding to those shown in Figure 1d due to the three distances.

To verify diffusion enhancement within the gel constructs induced by oscillation of the embedded iron microparticles, 0.5 μL of a water-based ink (LP2RF-8UF-L, Pilot Corporation, Tokyo, Japan) was injected at the interface between the basement layer and the upper test gel layer. The sample dish was then placed on the stage of the sweeping device at varying distances (9.0, 10.0, or 12.0 mm) above the coin magnets. A dynamic magnetic field with a sweeping frequency of 0.5 Hz was applied for up to 24 h at room temperature. Figure 2a shows representative time-lapse images of ink diffusion within the collagen gels. Qualitatively, oscillatory motion of the embedded iron microparticles markedly enhanced ink diffusion when the samples were positioned at distances of 9.0 or 10.0 mm above the magnets, whereas diffusion enhancement at a distance of 12.0 mm was less apparent visually. However, quantitative evaluation of diffusion via image processing revealed that the relative staining intensity at the ink injection sites decreased significantly at all three distances compared with no-particle controls (Figure 2b).

Notably, for samples positioned 9.0 mm above the magnets, the diffusion enhancement effect was unstable (triangular markers in Figure 2b). This instability arose because the magnetic field intensity at this distance was sufficiently strong to extract iron microparticles from the collagen gel, leading to particle aggregation and migration to the bottom of the culture dish, as indicated by red arrows in the left column of Figure 2a. Consequently, experiments at the 9.0 mm distance were terminated after 8 h.

To further quantitatively evaluate the diffusion enhancement, the temporal change in staining intensity shown in Figure 2b was interpreted as a proxy for ink concentration decay due to diffusion. A theoretical model, described in the Methods section and Appendix A, was applied to characterize the diffusion process. This model predicts that the concentration at the initial ink injection site decays as the sum of a fast and a slow exponential component:

(1) $$c(0,t)=c_{\infty }+(1-c_{\infty })(Be^{-\alpha_{f}t}+(1-B)e^{-\alpha_{s}t})$$

where *α_f_* and *α_s_* are the rate constants corresponding to the fast and slow decay processes, respectively (*α_f_* > *α_s_*), *c_∞_* represents the final concentration at the uniform steady state, and *B* denotes the fractional contribution of the fast decay component.

The solid curves in Figure 2b represent theoretical fits to Equation (1) for each experimental condition, and the fitted parameters are summarized in Table 1. For the control samples, representing the baseline condition of purely thermal diffusion, the fast decay parameter *α_f_* was close to the theoretical prediction (on the order of 10^−4^ s^−1^, see Appendix A), whereas the slow decay parameter *α_s_* was approximately two orders of magnitude smaller than the theoretical value (on the order of 10^−5^ s^−1^). This discrepancy is attributed to the diffusion barrier imposed by the collagen network. In contrast, oscillation of the embedded iron microparticles markedly enhanced diffusion within the gels, as evidenced by significant acceleration of both the fast and slow decay components, a reduction in *c*_∞_, and an increased contribution of the fast decay component. Because diffusion enhancement was pronounced at the sample distance of 10.0 mm while the iron microparticles retained stably within the gel, this distance condition was selected for all subsequent cell culture experiments.

### 2.2. Effects of Iron Particle Oscillation on Embedded Cells

**Figure 3:**
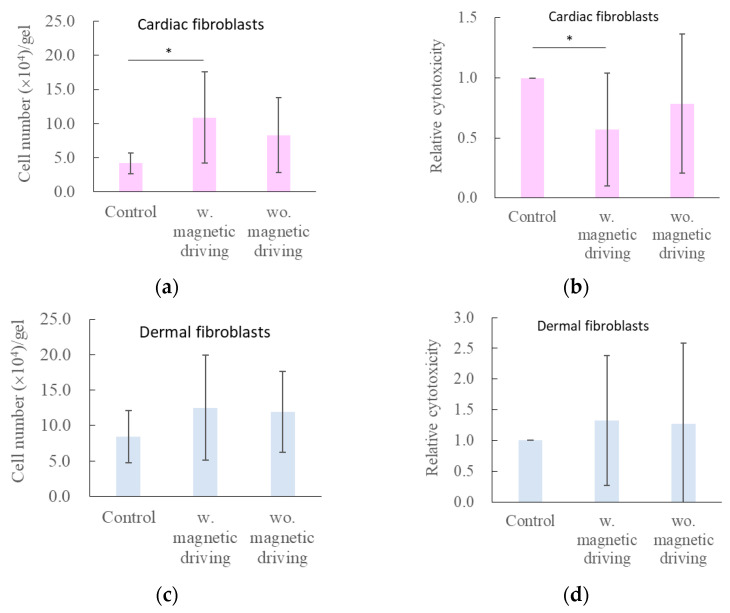
Effects of iron particle oscillation in collagen gels on embedded cells (data are plotted as mean ± SD; Four and three independent experiments were conducted for cardiac and dermal fibroblasts, respectively, with n = 3–4 samples per condition in each experiment). Oscillation significantly enhances cardiac fibroblast proliferation (**a**) and reduces cytotoxicity (**b**), whereas no significant effects are observed in dermal fibroblasts (**c**,**d**). The asterisks indicate significant differences (*p* < 0.05).

Figure 3 summarizes the effects of iron particle oscillation on cells embedded within collagen gels. In cardiac fibroblast cultures (Figure 3a,b), particle oscillation significantly enhanced cellular proliferation and reduced relative lactate dehydrogenase (LDH) release, indicating improved cell viability. In control samples, the number of cardiac fibroblasts decreased from the initial seeding density of 5.0 × 10^4^ cells to approximately 4.2 × 10^4^ cells per sample. In contrast, iron particle oscillation induced a significant, approximately twofold increase in cell number relative to the control. Interestingly, samples containing iron particles without magnetic actuation also exhibited an increase in cell number to 8.3 × 10^4^ cells; however, this increase did not reach statistical significance.

In contrast to cardiac fibroblasts, the effects of iron particle oscillation on dermal fibroblasts were limited. As shown in Figure 3c,d, no significant enhancement of proliferation was observed in samples containing iron particles, either with or without oscillation. Notably, dermal fibroblasts in control samples proliferated to approximately 8.4 × 10^4^ cells, whereas cardiac fibroblasts exhibited a net decrease under the same control conditions. These results underscore distinct proliferative responses of fibroblasts derived from different tissue sources within 3D collagen matrices.

### 2.3. Effects of Microtube Actuation on Dermal Fibroblasts

**Figure 4:**
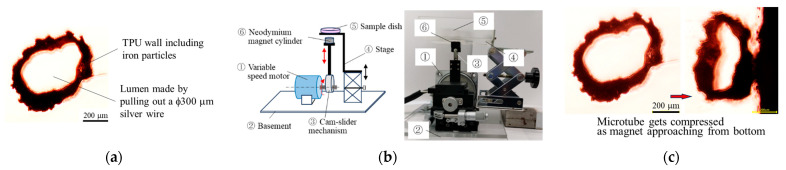
(**a**) Photograph of a polyurethane microtube with attached iron particles. (**b**) Schematic illustration and photograph of the linear device used to apply a cyclic dynamic magnetic field to culture gels placed on the stage. Red arrows indicate the direction of movement. (**c**) Representative photographs showing elastic compression of the microtube as it approaches a magnet at a distance corresponding to the thickness of the dish bottom.

Figure 4a shows a representative microtube (hereafter referred to as a μTube), consisting of a polyurethane wall decorated with gold-plated iron particles. The inner diameter of the μTubes was defined by fabrication around a 0.3 mm-diameter stainless steel wire, and the tube length was trimmed to 0.5 mm. To actuate the embedded μTubes, a second custom-built magnetic actuation system—termed the “linear device”—was developed (Figure 4b; Supplementary Video SI-v3).

As shown in Figure 4c, the cross-sectional area of a μTube decreased to approximately 50% of its original value when the magnet approached to within 3.0 mm, which represented the minimum practical distance achievable with the linear device under the experimental conditions. Recoil of the microtube diameter upon removal of the magnet was also confirmed. For cell culture experiments, three μTubes were aligned in series and embedded within each upper-test collagen gel. Dermal fibroblast cultures were maintained under these conditions for one week.

**Figure 5:**
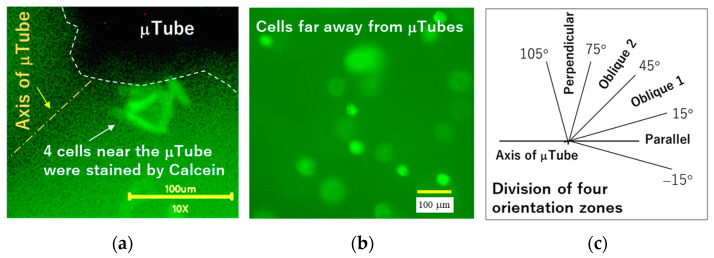
Fluorescence micrograph showing stained dermal fibroblasts near (**a**) or away (**b**) from μTubes. The alignment of three embedded μTubes was regulated using a 0.1 mm diameter platinum wire threaded through them, which defined the microtube axis. Cells exhibit fluorescence following Calcein-AM staining. (**c**) Illustration of the division of four cellular orientation zones, parallel, oblique 1, oblique 2, and perpendicular, relative to the microtube axis.

As shown in Figure 5, cells were stained with Calcein-AM and visualized using an inverted microscope after the culture period. Five to six regions located either near the μTubes (Figure 5a) or far from the μTubes (Figure 5b) were randomly selected. Within each region, cell number, cell area, cell aspect ratio, and cell orientation relative to the μTube axis (Figure 5c) were quantified for all cells.

**Figure 6:**
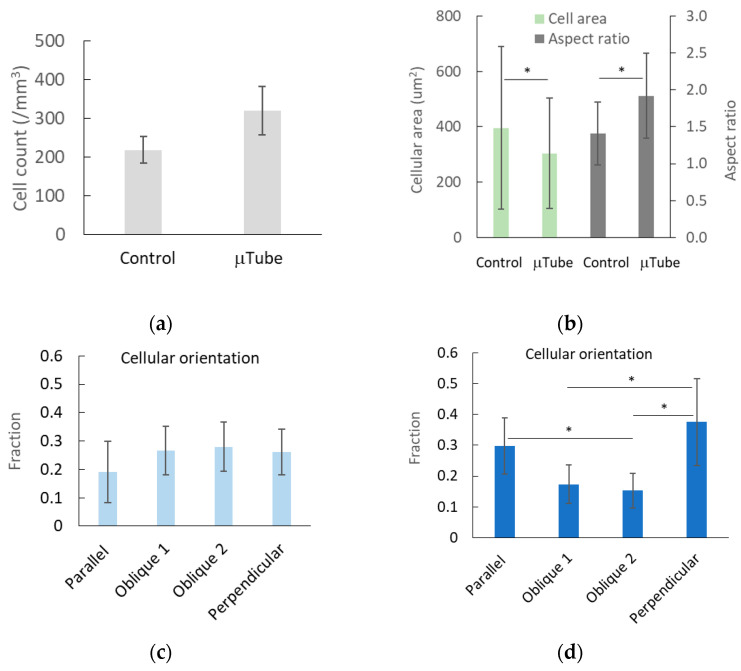
Effects of elastic compression of microtubes in collagen gels on embedded dermal fibroblasts. No significant proliferation was detected ((**a**); n = 3); instead, the primary effects were observed in cellular morphological changes (**b**) and orientation preferences of cells located near the microtubes (**d**), compared with cells located farther from the microtubes ((**c**), control). Panels (**b**), (**c**), and (**d**) were obtained from a single sample subjected to the cyclic dynamic magnetic field. Five to six random locations, comprising approximately 150 cells, were analyzed for regions near the microtubes and for regions farther away (control), respectively. The asterisks indicate significant differences (*p* < 0.05).

Figure 6 summarizes the effects of microtube actuation on dermal fibroblasts embedded within collagen gels. As shown in Figure 6a, cell number exhibited an increasing trend under microtube actuation but did not reach statistical significance. In contrast, fibroblasts located in proximity to the microtubes displayed a significant reduction in cell area and a significant increase in cellular aspect ratio, indicating a transition toward smaller and more elongated morphologies (Figure 6b). Analysis of cellular orientation (Figure 6c,d) revealed that fibroblasts located far from the microtubes exhibited no preferred orientation, whereas cells in close proximity to the microtubes showed a pronounced orientation bias, aligning either parallel or perpendicular to the microtube axis. Collectively, these observations suggest that local mechanical cues—potentially arising from interstitial flow and matrix deformation induced by microtube actuation—can regulate the morphology and alignment of embedded dermal fibroblasts.

### 2.4. Discussion

Three-dimensional (3D) cultures and tissue-engineered constructs face a fundamental limitation in delivering sufficient nutrients to, and removing metabolic waste from, their inner regions [3,4,5,19]. The most direct and biomimetic solution to this challenge is the formation of capillary-like, perfusable networks within the 3D structure. To date, a variety of bottom-up strategies have been developed to address this goal. One approach involves subdividing large constructs into smaller, perfusable compartments, each equipped with microchannels to support cell culture; microfluidic platforms are representative of this category [20,21,22]. Another widely adopted strategy employs three-dimensional bioprinting technologies to fabricate vascular networks directly or to integrate prefabricated tubular components during the printing process [23,24,25].

Despite differences in fabrication techniques, these systems typically rely on a single external pump to maintain perfusion throughout the 3D construct [26,27]. Such configurations suffer from inherent limitations, particularly in achieving uniform and stable perfusion across the entire structure. These limitations become more pronounced during long-term culture, as cellular activity induces structural remodeling that can disrupt or even obstruct flow distribution [26,27,28].

Recently, magnetically responsive hydrogels have emerged as a promising class of smart biomaterials for biomedical applications [29,30,31,32,33]. These materials respond to externally applied magnetic fields, enabling remote modulation of the physical, biochemical, and mechanical properties of the cellular microenvironment. However, most previous studies have focused on macroscopic changes in hydrogel properties or on direct interactions between magnetic particles and embedded cells to regulate cellular behavior. The use of magnetic actuation specifically to enhance mass transport within 3D constructs remains comparatively underexplored.

In light of these challenges and advances, we propose an alternative and complementary strategy to address perfusion limitations in 3D cell constructs by incorporating internally distributed driving sources within the hydrogel matrix. In this proof-of-concept study, two types of magnetic components were embedded into collagen hydrogels to actively enhance diffusion under dynamic magnetic fields.

In this study, cardiac and dermal fibroblasts were selected as representative mesenchymal cell types that share common extracellular matrix interactions yet differ in their sensitivities to mass transport and mechanical cues. Both cell types actively interact with type I collagen, making them well suited for 3D collagen gel culture. Cardiac fibroblasts are known to exhibit higher metabolic demand and greater sensitivity to diffusion limitations and interstitial flow, whereas dermal fibroblasts are comparatively robust under different conditions. This contrast enables the effects of enhanced diffusion within the gel to be highlighted on tissue-specific cellular responses to transport-mediated cues.

The results demonstrate clear cell type–dependent responses to iron particle oscillation. In experiments using gold-plated iron particles (Figure 3), cardiac fibroblasts—whose proliferation was initially suppressed in collagen gels—exhibited a significant increase in cell number under dynamic magnetic stimulation, accompanied by a marked reduction in cytotoxicity (Figure 3a,b). In contrast, dermal fibroblasts, which proliferated readily in collagen gels under control conditions, exhibited limited responses to particle oscillation. Although a slight increase in proliferation was observed, it did not reach statistical significance, and cytotoxicity showed a tendency to increase.

Fibroblasts originate from the embryonic mesenchyme and subsequently differentiate into tissue-specific subtypes that reside in virtually all tissues, where they typically remain in a quiescent state. Once harvested and cultured in vitro, fibroblasts become activated and generally exhibit a markedly higher proliferation rate than in vivo. Nevertheless, their tissue-specific characteristics remain partially imprinted despite in vitro culture conditions [34]. Recent studies have identified two major universal fibroblast subtypes, with dermal and cardiac fibroblasts belonging to distinct categories [35,36].

Cardiac fibroblasts have been reported to consume more oxygen in monolayer culture than other mesenchymal cells, such as smooth muscle cells and endothelial cells [37]. They are also highly sensitive to variations in oxygen tension. For example, cardiac fibroblasts exhibit substantially greater mitotic activity under conventional culture oxygen levels compared with the much lower physiological oxygen tension present in vivo [38]. Furthermore, hypoxic conditions have been shown to increase the expression of hypoxia-inducible factor-1α (HIF-1α) in cardiac fibroblasts, which suppresses proliferation and promotes a shift toward glycolytic metabolism [39]. In contrast, dermal fibroblasts demonstrate greater tolerance to hypoxia, which may largely reflect their physiological role in wound healing in vivo. Accordingly, dermal fibroblasts are able to maintain proliferation and may even exhibit enhanced metabolic activity in three-dimensional collagen gel cultures under low-oxygen conditions [40].

With regard to glucose uptake, glucose represents the predominant energy source under standard culture conditions. Glucose transporter isoform 1 (GLUT1) is the principal transporter expressed in both dermal and cardiac fibroblasts. However, GLUT1 expression in cultured dermal fibroblasts appears to exhibit more dynamic regulation than in cardiac fibroblasts [41,42], suggesting superior metabolic adaptability of dermal fibroblasts to environmental changes.

Taken together, the most plausible explanation for the distinct proliferative behaviors observed in this study is the higher metabolic demand but lower environmental adaptability of cardiac fibroblasts compared with dermal fibroblasts in culture. Therefore, in dense collagen gels, diffusion limitations of oxygen and nutrients are likely to affect cardiac fibroblasts more severely. Under such conditions, diffusion enhancement would preferentially improve the proliferation of cardiac fibroblasts while exerting less effects on dermal fibroblasts.

In addition to metabolic factors, cellular mechanosensitivity may also contribute to the observed differences. Cardiac fibroblasts are highly mechanosensitive and normally reside in the stiffer, load-bearing myocardium (~10–20 kPa). Consequently, they may enter quiescence or undergo apoptosis in soft 3D collagen gels (~0.5 kPa for 2 mg/mL collagen) under the control conditions. Dermal fibroblasts, by contrast, are adapted to the mechanically heterogeneous skin environment and tolerate softer matrices more effectively while maintaining matrix tension. Furthermore, iron particle actuation within the gels may provide direct mechanical stimulation to mechanosensitive cardiac fibroblasts, partially offsetting the adverse effects of the soft collagen matrix [43,44].

The effects of microtube actuation were primarily manifested as changes in cellular morphology and orientation in regions proximal to the microtubes (Figure 6). Morphological analysis revealed that cells located near the magnetically actuated microtubes underwent significant remodeling, characterized by reduced cell area, increased aspect ratio, and pronounced orientation bias (Figure 6b,d). This alignment behavior can be attributed to two primary biophysical cues: topographical guidance and fluid shear stress. Previous studies have demonstrated that magnetically responsive nanostructures or nanoparticles can form anisotropic physical scaffolds under magnetic fields, thereby directly guiding cellular orientation and cytoskeletal organization [32,33]. However, in the present system, the dynamic cyclic compression–recoil motion of the microtubes suggests an alternative—and likely dominant—mechanism: the generation of interstitial fluid flow.

Actuation of the microtubes is expected to displace the surrounding hydrogel matrix and culture medium, creating localized and dynamic fluid flow within the 3D interstitial space. It is well documented that many cell types, including fibroblasts and endothelial cells, reorganize their cytoskeleton and orient their cell bodies in response to fluid shear stress. A classic response, particularly for endothelial cells in monolayers subjected to unidirectional laminar flow, is alignment parallel to the flow direction, which minimizes shear stress across the cell body [45,46]. In contrast, in more complex 3D microenvironments or under oscillatory flow conditions, cellular responses are more variable. Fibroblasts embedded within 3D matrices, for example, have been reported to align perpendicular to the dominant flow direction, potentially due to strain-induced matrix reorganization or direct mechanotransduction of drag forces [47,48].

Given the periodic compression–recoil motion of the microtubes, the induced interstitial flow in the present system is unlikely to be purely unidirectional. Instead, it is expected to consist of oscillatory or radially expanding and contracting flow fields. Cells embedded within fibrillar 3D matrices are highly sensitive to such flow-mediated matrix deformations. The observed orientation bias is therefore most parsimoniously explained by interstitial flow–mediated effects, in which cyclic fluid motion generates shear stress and induces microscale strains in the pericellular matrix. These mechanical cues, in turn, activate integrin-based mechanosensing pathways and downstream signaling cascades—such as those involving Rho GTPases and actomyosin contractility—that drive cytoskeletal remodeling and cellular reorientation [49,50,51].

As a proof-of-concept investigation, this study has certain limitations, particularly the lack of comprehensive metabolic characterization of the cultured cells. Specifically, to elucidate the divergent responses to iron particle oscillation observed between cardiac and dermal fibroblasts, detailed physicochemical analyses of oxygen and glucose consumption are required and are planned for future work. In this regard, time-course experiments (e.g., measurements performed daily), rather than the current end-point assessments, would provide more robust and convincing evidence. Therefore, time-course experiments will be conducted to monitor oxygen and glucose concentrations in the microenvironment surrounding the embedded cells, as well as the expression levels of HIF-1α and GLUT1.

Another limitation is the potential generation of oxidative stress in the microtube experiments arising from clusters of iron particles attached to the polyurethane microtubes, particularly in cases where gold plating may have been incomplete. In the experiment, the total mass of attached iron particles was estimated to exceed 10 mg across three microtubes, and yellowing of the culture medium was observed during the final two days of culture. Oxidative stress induced by iron particles has been reported previously [52,53]. Although catalase supplementation was employed as a countermeasure, it may not have been sufficient to fully mitigate iron-mediated oxidative effects [54,55]. This limitation may partially account for the absence of significant dermal fibroblast proliferation observed in the microtube-driven experiments.

In future studies with microtubes, we will first replace carbonyl iron particles with iron oxide–based magnetic materials during microtube fabrication to further reduce oxidative stress. We will then focus on direct measurement of interstitial flow in the vicinity of actuated microtubes, investigation of the underlying cellular signaling pathways, and evaluation of endothelial cell angiogenesis and migration. Together, these efforts aim to develop the proposed internally powered actuation strategy into a robust and effective modality for engineered tissue construction.

## 3. Conclusions

This proof-of-concept study demonstrates that dynamic magnetic actuation of embedded iron particles and microtubes can function as internally distributed driving sources to enhance diffusion and perfusion within three-dimensional constructs. The observed improvement in cardiac fibroblast viability and the pronounced morphological and alignment changes of dermal fibroblasts underscore the potential of this approach to augment metabolic transport and provide biophysical cues relevant to angiogenesis in hydrogel-based, tissue-engineered systems.

## 4. Materials and Methods

### 4.1. Cell Preparation

Dermal and cardiac fibroblasts were harvested from Wistar rat neonates. All procedures in animal experiments followed the Guide for the Care and Use of Laboratory Animals of the National Institutes of Health, Bethesda, MD, USA, and were approved by the Animal Studies Ethics Committee of Yamagata University. Primary culture for each type of these cells was last for a week and passages followed. Cardiac fibroblasts through one or two passages and dermal fibroblasts through two or three passages were used in experiment. The details of cellular harvest and culture may refer [56].

### 4.2. Test Platforms Composed of Collagen Gels and Iron Microparts

Each test platform (Figure 1a) was fabricated through a two-step procedure as explained in details as follows:

Step 1: formation of Basement Gel

Collagen type I solution (3.60–4.10 mg/mL in 0.02 N acetic acid, Corning) was diluted to 1.5 mg/mL with phosphate-buffered saline (PBS) and neutralized using 0.1 M NaOH. A 0.5 mL aliquot of this solution was poured into silicone molds (10.0 mm width × 25.0 mm length × 5.0 mm thickness) and incubated for 1 h in a CO_2_ incubator to form a basement gel layer approximately 2.0 mm thick. In some experiment as indicated later gels were formed in wells of 24-well plates instead of the silicone molds.

Step 2: formation of Test Gel Layer

A second 0.5 mL aliquot of collagen solution (1.5 mg/mL) containing magnetic microparts (described below) and rat dermal or cardiac fibroblasts (5.0 × 10^4^ cells) was layered on top of the basement gel. This two-step procedure was adopted to prevent sedimentation of the iron microparts at the bottom, thereby positioning them near the center of the gel entire thickness and enabling more reliable assessment of internal diffusion enhancement.

### 4.3. Two Types of Microparts Were Embedded in the Gels

(1) Gold-plated iron particles:

Carbonyl iron microparticles (Qinghe County Chuangying Metal Materials Co., Ltd., Xingtai, China) were coated with approximately 0.2 μm thick gold plating (Kiyokawa Plating Industry Co., Ltd., Fukui, Japan) to prevent oxidation during cell culture. The average diameter of the plated particles was 50.0 ± 7.0 μm.

A 2.0 mg quantity of particles was added to the collagen solution when forming the upper test gel layer. As shown in Figure 1b, the distribution of particles was observed using an inverted microscope (IX71, Olympus, Tokyo, Japan) focused at the basement–upper gel interface. The particles were found to be dispersed throughout the thickness of test gels, though most of the particles sedimented at the interface. The inter-particle spacing was estimated at ~200 μm.

(2) Iron-particle-included microtubes:

Microtubes (also indicated as μTubes) were fabricated by dissolving 1.0 g of thermoplastic polyurethane (TPU; LS86W2, DIC Covestro Polymer Ltd., Tokyo, Japan) in 10.0 mL tetrahydrofuran (THF; Tokyo Chemical Industry Co., Ltd., Tokyo, Japan). A 0.3 mm diameter stainless steel wire was dipped into this TPU-THF solution, then rolled in gold-plated iron particles to adhere them to the film. After solvent evaporation, microtubes with particle-attached TPU walls were peeled off the wire and cut into 0.5 mm lengths (Figure 4a).

These microtubes were designed to oscillate radially under an external magnetic field—compressing under magnetic force and recoiling via elastic restoration—to function as microscale pumps. In this study, three such microtubes were aligned and suspended in each gel using a thin platinum wire (0.1 mm diameter), which also functioned to stabilize the microtubes in gels and regulated the axes of the microtubes.

### 4.4. Magnetic Actuation of Iron Particles and Diffusion Enhancement Mechanism

To induce oscillatory motion of the embedded iron particles, a custom-built device (Figure 1c; Supplementary Video SI-v1), termed the “sweeping device,” was used. It employed a variable-speed motor to drive a cam mechanism, which reciprocally swept an arm of an acrylic strip holding three neodymium magnets beneath the sample stage. Each magnet had a surface magnetic field of 350 mT. The sweeping frequency was adjustable between 0.5 and 5.0 Hz. The vertical distance between the magnets and sample dish was modulated by inserting different numbers of 1.0 mm thick silicone sheets between the stage and the dish. On the stage there was either a dish containing a test gel in the silicone mold or a 24-well plate including multiple gels in wells as explained later.

The magnetic field strength on the stage surface was measured using a Tesla meter (MG-801, Magna Co., Ltd., Tokyo, Japan), and field dynamics were recorded via video and quantified as shown in Figure 1d. To verify particle oscillation, movement of embedded iron particles in collagen gels was observed under an inverted microscope (IX71, Olympus, Japan) during magnetic actuation. With the magnets positioned 10.0 mm above the sample, particle oscillating magnitude was measured at 7.0 ± 5.0 μm (n = 10), as shown in Supplementary Video SI-v2.

To elucidate the mechanism underlying diffusivity enhancement by the oscillating embedded iron particles, we modeled the diffusion of the injected ink as a two-dimensional process within a circular disk. This analysis led to the approximate Equation (1), which describes the temporal decay of the ink concentration at the injection site as a superposition of fast and slow exponential components, ultimately converging to a uniform steady state (see Appendix A for details).

For fitting the experimental data to Equation (1), a data-determination algorithm combined with manual adjustment, as described in Ref. [57], was employed to extract the parameter values listed in Table 1.

### 4.5. Actuation of Microtubes

To drive the embedded microtubes, another custom-built device—termed the “linear device”—was developed (Figure 4b; Supplementary Video SI-v3). Using a cam-slider mechanism, a neodymium magnetic cylinder (surface field: 480 mT) was driven to approach and recede from the bottom of the sample dish. This generated a dynamic magnetic field that proved effective at inducing radial deformation of the microtubes. As demonstrated in Figure 4c, the cross-sectional area of a microtube decreased to ~50% of its original value when the magnet approached within 3.0 mm—the practical distance achieved with this device in the experiment. The frequency of magnetic oscillation was adjustable between 0.5 and 5.0 Hz.

### 4.6. Three Experiments Conducted in This Study

#### 4.6.1. Experiment 1: Diffusion Enhancement by Oscillating Iron Microparticles

The first experiment aimed to verify diffusion enhancement in the gel constructs due to embedded iron microparticles. No cells were included in this experiment. Instead, 0.5 μL of water-based ink (LP2RF-8UF-L, Pilot Corporation, Tokyo, Japan) was injected at the interface between the basement and test gel layers (Figure 2a). The sample dish was placed on the stage of the sweeping device at varying heights above the magnetic coins. A dynamic magnetic field with a sweeping frequency of 0.5 Hz was applied for 24 h at room temperature.

Images of the samples were captured at 0, 1, 2, 3, 4, 6, 8, and 24 h. Ink diffusion was quantified using ImageJ 1.54g (https://imagej.net/ij/) as follows:

(1) Each image was converted to an 8-bit grayscale format. (2) The mean gray value within a fixed region of the ink stain was measured. (3) Background gray value from a non-stained region of the gel was subtracted to obtain the contrast mean gray value.

Diffusion was expressed as a relative staining ratio, calculated by dividing the contrast gray value at each time point by that of the initial image (immediately after ink injection, before magnetic field application).

#### 4.6.2. Experiment 2: Effect of Oscillating Iron Particles on Fibroblasts Culture

The second experiment investigated the impact of oscillating iron particles on embedded dermal or cardiac fibroblasts. Fibroblasts (5.0 × 10^4^ cells) were mixed into each test gel along with 2.0 mg of gold-plated iron particles. To mitigate oxidative stress from incompletely plated particles, catalase (Wako, Osaka, Japan) at final concentration of 18.0 μg/mL was supplemented in the gel during sample formation.

In this experiment three conditions for the gels were tested: control gels without iron particles, particle-still gels with iron particles embedded but without magnetic field imposed, and particle-oscillating gels with iron particles embedded under sweeping magnetic field. Four gels for each condition were formed in four wells of a 24-well plate instead of silicone molds in each experiment. The plate was placed on the stage 10.0 mm above the sweeping magnetic arm of the device as shown in Supplementary Video SI-v1. Culture was conducted in a 5% CO_2_ incubator where the sweeping device was installed. Gels were immersed in 1.2 mL of culture medium (high-glucose DMEM supplemented with 10% fetal bovine serum, 1% penicillin–streptomycin, and 9.0 μg/mL catalase). Culture lasted for 7 days, with medium changes on day 1 and day 4.

Cytotoxicity was assessed using an LDH cytotoxicity assay kit (DOJINDO, Tokyo, Japan). Culture medium collected on day 7 was analyzed following the manufacturer’s protocol.

Absorbance (optical density, OD) at 490 nm was measured using a microplate reader (MPR-A100, AS ONE, Tokyo, Japan). Average values from four wells were calculated, and background absorbance (from unused culture medium) was subtracted. The data were first evaluated within the reasonable range for an effective measurement obtained through a preliminary experiment with monolayer culture in a 48-well plate. The final results were expressed as relative cytotoxicity ratio to the control measurements.

Cell proliferation was quantified using the CyQuant NF Kit (Thermo Fisher Scientific, Tokyo, Japan), according to the manufacturer’s protocol. Gels were washed with PBS and dissolved in 1.5 mL of 0.02 N acetic acid in PBS. Iron particles were allowed to sediment, and the supernatant containing cells was collected and resuspended in CyQuant NF buffer. Fluorescence was measured using a microplate reader (excitation ~485 nm, emission ~530 nm; MPR-A100, AS ONE). Cell number was calculated by interpolation of the fluorescence intensity against a pre-obtained linear relationship between cell number and fluorescence intensity.

#### 4.6.3. Experiment 3: Effect of Microtubes on Dermal Fibroblast Culture

The third experiment evaluated the effects of embedded microtube structures using the linear magnetic field device (Figure 4b). Instead of iron particles, three microtubes were aligned in series and embedded in each collagen gel. Test sample was placed on the stage and the magnet was adjusted to approach the sample within 3.0 mm including thickness of the sample dish bottom. For evaluation of the culture, the gels were fluorescence-stained by Calcein-AM (DOJINDO, Tokyo, Japan). Cell count and morphological measurements were conducted on the photographs taken by the Olympus inverted microscope. Calcein-AM staining followed the manufacturer’s instructions: Gels were rinsed with 1.0 mL of PBS and then immersed in 1.0 mL of culture medium containing 0.2% Calcein-AM. After 30 min of incubation, the gels were rinsed with PBS and re-immersed in fresh culture medium. Cells were visualized using the inverted microscope. Five to six locations either near or far from the microtubes were randomly selected, and cell count, cell area, and cell aspect ratio were quantified for all cells within these regions. The cell aspect ratio was defined as the length of the cellular long axis divided by the longest length perpendicular to that axis. In addition, cell orientation relative to the microtube axis was measured and categorized into four directions to the microtube axis: parallel (−15~15°), oblique 1 (15~45°), oblique 2 (45~75°), and perpendicular (75~105°), as illustrated in Figure 5c. All cellular morphological measurements were performed using ImageJ.

### 4.7. Statistical Analysis

For comparisons between two groups, Student’s t-test was used. For comparisons among multiple groups, non-parametric one-way ANOVA was performed, followed by the Tukey post hoc test. A *p*-value of less than 0.05 was considered statistically significant.

## Figures and Tables

**Table 1:** Diffusion parameters obtained by fitting the experimental data to Equation (1).

	Control	12.0 mm Above	10.0 mm Above	9.0 mm Above
*c*_∞_	0.45 ± 0.083 ^ab^	0.33 ± 0.031	0.25 ± 0.03 ^a^	0.23 ± 0.11 ^b^
*α_f_*	(1.1 ± 0.55) × 10^−4 ab^	(1.8 ± 0.25) × 10^−4 cd^	(3.7 ± 0.87) × 10^−4 ac^	(4.2 ± 3.2) × 10^−4 bd^
*α_s_*	(5.0 ± 4.0) × 10^−7 ab^	(7.8 ± 2.3) × 10^−7 cd^	(8.2 ± 1.8) × 10^−6 ace^	(5.4 ± 3.2) × 10^−5 bde^
*B*	0.18 ± 0.019 ^ab^	0.36 ± 0.059 ^cd^	0.54 ± 0.037 ^ac^	0.56 ± 0.15 ^bd^

The data pairs with the same superscript letter in each row are significantly different (*p* < 0.05).

## Data Availability

The datasets generated during and/or analyzed during the current study are available from the corresponding authors on reasonable request.

## Supplementary Materials

The following supporting information can be downloaded at: https://www.mdpi.com/article/10.3390/gels12030225/s1, Video SI-v1: Operation of the sweeping device; Video SI-v2: Iron particle oscillation under cyclically dynamic magnetic field; Video SI-v3: Operation of the linear device.

## Appendix A

To elucidate the mechanism of diffusivity enhancement induced by oscillating embedded iron particles, we analyzed the surrounding fluid motion generated by particle oscillation using the classical model of a sphere oscillating in a viscous medium, as formulated in Refs. [58,59,60]. Such oscillation generates local perturbation flow confined within the Stokes boundary layer, whose characteristic thickness is estimated as:

(A1) $$\delta =\sqrt{\frac{2\nu }{\omega }}$$

where *δ* denotes the boundary-layer thickness that characterizes the spatial region affected by particle vibration; *ν* is the kinematic viscosity of medium; and *ω* is the angular frequency of particle oscillation.

Oscillatory actuation of microparticles introduces mechanical energy into the surrounding medium, generating local velocity fluctuations and microstructural rearrangements. These perturbations act as an additional stochastic forcing on nearby diffusing molecules, thereby increasing their mean square displacement and enhancing the effective diffusion coefficient.

For baseline thermal diffusion, the mean square displacement of a diffusing molecule is given by:

(A2) $$\Delta_{T}^{2}=2dD_{0}t,\;\;D_{0}=\frac{k_{B}T}{\varsigma }$$

where $\Delta_{T}^{2}$ denotes the mean square displacement, *D*_0_ is the diffusion coefficient; *d* is the spatial dimensionality, *t* is time; *k_B_* is the Boltzmann constant; *ς* is the drag friction of diffusion particles in medium; and *T* is the absolute temperature. The diffusion coefficient is determined by the Stokes-Einstein relation [61]:

(A3) $$D_{0}=\frac{k_{B}T}{6\pi \eta \gamma }$$

where *η* is the dynamic viscosity of the medium and *γ* is the radius of diffusion molecules.

When an oscillating microparticle is present, it generates a time-dependent velocity field within the Stokes boundary layer *δ*, which contributes an additional component to the molecular displacement. The resulting effective mean square displacement can be expressed as [62,63,64]:

(A4) $$\Delta_{eff}^{2}=\Delta_{T}^{2}+\Delta_{a}^{2}$$

where Δ*_eff_* represents the total effective displacement, Δ*_T_* arises from thermodynamic motion; and Δ*_a_* arises from particle-induced perturbation. Consequently, the effective diffusion coefficient increases relative to purely thermal diffusion and can be expressed analogously to Equation (A2) as:

(A5) $$\Delta_{eff}^{2}=2dD_{eff}t,\;\;D_{eff}=\frac{k_{B}T}{\varsigma }$$

To model the experimentally observed ink diffusion, the ink stain was treated as undergoing two-dimensional diffusion within a circular disk of radius *R*, with an initial narrow Gaussian concentration profile. According to Fick’s second law, the concentration *c*(*r*,*t*) evolves as a function of radial distance *r* and time *t* [65]:

(A6) $$\frac{\partial c(r,t)}{\partial t}=D\frac{1}{r}\frac{\partial }{\partial r}(r\frac{\partial c(r,t)}{\partial r}),\;\;0\leq r\leq R$$

where *D* denotes diffusion coefficient, and the no-flux boundary condition at *r* = *R* is

(A7) $${\left.\frac{\partial c}{\partial r}\right|}_{r=R}=0$$

For a narrow Gaussian initial ink injection at the disk center, the initial condition is

(A8) $$c(r,0)=\exp(-\frac{r^{2}}{2\sigma^{2}}),\;\;\sigma \ll R$$

where *σ* is the standard deviation, and the following normalization is applied

(A9) c(0,0)=1

Applying separation of variables,

(A10) $$c(r,t)=\sum_{k}a_{k}e^{-D\lambda_{k}^{2}t}\phi_{k}(r)$$

and substituting it into Equation (A6), we get

(A11) $$\frac{d^{2}\phi }{dr^{2}}+\frac{1}{r}\frac{d\phi }{dr}+\lambda^{2}\phi =0$$

This is a zero-order Bessel equation, yielding

(A12) $$\phi (r)=J_{0}(\lambda r)$$

where *J*_0_(*x*) denotes the zeroth-order Bessel function of the first kind.

Applying the boundary condition in Equation (A7) leads to

(A13) $${J^{\prime }}_{0}(\lambda R)=0,$$

Since ${J^{\prime }}_{0}(x)=-J_{1}(x)$, where *J*_1_(*x*) denotes the first-order Bessel function of the first kind, this implies $J_{1}(\lambda R)=0$; therefore, $\lambda_{k}=\mu_{k}/R$, where *μ_k_* are the zeros of *J*_1_(*x*).

Thus, the general solution is

(A14) $$c(r,t)=a_{0}+\sum_{k=1}a_{k}e^{-D\lambda_{k}^{2}t}J_{0}(\lambda_{k}r)$$

Using the orthogonality of Bessel functions, *a_k_* are obtained as

(A15) $$a_{k}=\frac{2}{R^{2}J_{0}^{2}(\lambda_{k}R)}\int_{0}^{R}c(r,0)J_{0}(\lambda_{k}r)rdr\;\;\;\;(k\geq 1)$$

and for *k* = 0,

(A16) $$a_{0}=\frac{2}{R^{2}}\int_{0}^{R}c(r,0)rdr$$

Substituting the narrow Gaussian initial condition (Equations (A8) and (A9)) yields

(A17) $$\begin{array}{ll} a_{k} & =\frac{2}{R^{2}J_{0}^{2}(\lambda_{k}R)}\int_{0}^{R}e^{-r^{2}/(2\sigma^{2})}J_{0}(\lambda_{k}r)rdr\approx \frac{2}{R^{2}J_{0}^{2}(\lambda_{k}R)}\int_{0}^{\infty }e^{-r^{2}/(2\sigma^{2})}J_{0}(\lambda_{k}r)rdr\\ & =\frac{2}{R^{2}J_{0}^{2}(\lambda_{k}R)}e^{-\lambda_{k}^{2}\sigma^{2}/2} \end{array}$$

The concentration evolution at *r* = 0 is then given by

(A18) $$c(0,t)=a_{0}+\sum_{k=1}a_{k}e^{-D\lambda_{k}^{2}t}$$

In this study, we approximate Equation (A18) by retaining only the first three terms and modify them as

(A19) $$c(0,t)=c_{\infty }+(1-c_{\infty })(Be^{-\alpha_{f}t}+(1-B)e^{-\alpha_{s}t})$$

This expression corresponds to Equation (1) in the main text, where *α_f_* and *α_s_* are the rate constants corresponding to the fast and slow decay processes, respectively (*α_f_* > *α_s_*), *c*_∞_ represents the final concentration at the uniform steady state, and *B* denotes the fractional contribution of the fast decay component. The parameter values were obtained by fitting experimental data.

For baseline thermal diffusion (control condition), *α_f_* and *α_s_* can be estimated as

(A20) $$\alpha_{f}=D_{0}{\left(\frac{\mu_{2}}{R}\right)}^{2}=D_{0}{\left(\frac{7.02}{R}\right)}^{2},\;\;\alpha_{s}=D_{0}{\left(\frac{\mu_{1}}{R}\right)}^{2}=D_{0}{\left(\frac{3.83}{R}\right)}^{2}$$

Using *T* = 300 K, *η* = 0.001 Pa.s, *γ* ≈ 1 × 10^−8^ m, and *R* ≈ 3 × 10^−3^ m; then *α_f_* and *α_s_* are estimated to be on the order of 10^−4^ s^−1^ and 10^−5^ s^−1^, respectively.
